# High Symptom Burden in Patients Receiving Radiotherapy and Factors Associated with Being Offered an Intervention

**DOI:** 10.3390/curroncol31030094

**Published:** 2024-02-27

**Authors:** Allison Rau, Demetra Yannitsos, Petra Grendarova, Siwei Qi, Linda Watson, Lisa Barbera

**Affiliations:** 1 Division of Radiation Oncology, University of Calgary, Calgary, AB T2N 4N1, Canada; allison.rau@albertahealthservices.ca (A.R.); demetra.yannitsos@albertahealthservices.ca (D.Y.); petra.grendarova@albertahealthservices.ca (P.G.); linda.watson@albertahealthservices.ca (L.W.); 2 Tom Baker Cancer Centre, Calgary, AB T2N 4N2, Canada; 3 Cancer Care Alberta, Calgary, AB T4N 4E7, Canada; siwei.qi@albertahealthservices.ca

**Keywords:** radiation oncology, radiation therapy, high symptom complexity, patient-reported outcomes

## Abstract

Patient report outcomes are commonly collected during oncology visits to elicit symptom burden and guide management. We aimed to determine the frequency of intervention for patients undergoing radiotherapy with high symptom complexity scores and identify which factors are associated with being offered an intervention. A retrospective chart audit was completed of adult patients with cancer who had at least one radiotherapy appointment and were assigned a high symptom complexity. A total of 200 patients were included; 150 (75.0%) patients were offered an intervention for the main symptom. The most offered intervention was medications. Multivariable logistic regression showed factors associated with being offered an intervention were the following: symptom score of 9 (OR = 9.56, 95% CI 1.64–62.8) and 10 (OR = 7.90, 95% CI 1.69–38.2); palliative intent radiation (OR 3.87, 96% CI 1.46–11.1); and last review appointment (OR 6.22, 95% CI 1.84–23.3). Symptoms associated with being offered an intervention included pain (OR 22.6, 95% CI 6.47–91.1), nausea (OR 15.7, 95% CI 1.51–412), shortness of breath (OR 7.97, 95% CI 1.20–63.7), and anxiety (OR 6.69, 95% CI 1.58–31.6). This knowledge will help guide clinical practice to understand symptom burden and how we can improve our management of patients’ symptoms.

## 1. Introduction

Experience of a person’s illness and treatment goes beyond clinical care and requires focus on individualised needs [1]. Understanding symptom burden and personal challenges can influence the experience of a person’s cancer journey [2]. Significant efforts have been made to better understand symptoms experienced from the patient’s perspective using patient-reported outcomes (PROs) in order to better address and support targeted and meaningful symptom management [3,4].

PROs are responses directly elicited from patients of the impact of the disease and treatment on their health and function [5]. The adoption of PROs has become an important part of clinical practice, particularly in oncological care [3,6]. The impacts of a cancer diagnosis and treatment can have significant physical and psychological effects on a person and their families, and PROs in cancer care have been adopted as a routine practice to help improve this experience [7,8].

Patient-reported outcome measures (PROMs) are instruments, typically in the form of questionnaires, developed to collect information on symptom burden, emotional well-being, and overall function [9,10]. PROMs help guide the clinician in addressing and supporting their patients throughout their disease and treatment journey. In a systematic review of PROMs in an oncologic setting, PROs were shown to have a positive impact on patient–clinician communication; improve the detection of unrecognised problems; improve the management of symptoms, treatment toxicity and side effects, and emotional well-being; and improve overall patient satisfaction [3,10,11,12,13].

PROMs can help to identify patients experiencing high symptom burden, recognizing that intervention for these symptoms will depend on the patient, tumour, and treatment-related factors. Earlier interventions and frequent monitoring of patients’ symptoms have been shown to improve patients’ quality of life, compliance, and engagement in more effective management strategies [14,15,16]. Although patients experiencing a higher symptom burden are thought to require close monitoring, there is minimal literature published about the management practices of patients with a higher symptom burden. There has been published literature that has shown patients with cancer and high symptom complexity are more likely to have an unplanned emergency visit [17]. The purpose of this study was to determine the frequency of intervention for patients with a high symptom burden and to understand which factors are associated with being offered an intervention for patients undergoing radiotherapy for cancer treatment.

## 2. Materials and Methods

### 2.1. Study Design 

A retrospective chart audit was completed of adult patients with cancer who had at least one radiotherapy appointment at a single tertiary cancer centre. We collected administrative data (unique identifier number, appointment type, and tumour type) from the provincial Alberta Cancer Registry (ACR), and the remaining data elements were collected from individual electronic medical records (EMR). We used this data to identify patients who had a high symptom complexity score. Data linkage was achieved through a unique provincial health care number assigned to each patient as part of the ACR’s process. This study is part of PROSE (Person-centered Radiation Oncology Service Enhancement), a quality improvement (QI) initiative that was developed to improve patient experience at a tertiary cancer centre in the radiation oncology department. The initiative has received ethics approval from the Health Research Ethics Board of Alberta’s Cancer Committee (HREBA.CC-20-0022). 

### 2.2. Study Sample

The study cohort included adult patients with a cancer diagnosis who were 18 years of age and older and who had at least one radiation therapy (RT) treatment at a tertiary cancer centre between 1 October 2019, and 1 April 2020. We originally intended to include patients from the entire province, but after review, 71.4% of all the records came from one centre, so we restricted the study to this centre only. To be included in this patient cohort, patients must have completed a PROs questionnaire within this timeframe and have been found to have a high symptom complexity score. 

### 2.3. Measurements 

The Putting Patients First (PPF) form is a PROM that is routinely collected across the province. At our institution, the PPF is collected at consultation appointments, first and last treatment review appointments, and follow-up appointments. For the purpose of this study, we focused only on PPFs completed at consultation appointments and treatment review appointments. The PPF consists of 2 parts: the revised Edmonton Symptom Assessment Scale (ESAS-r) and the Canadian Problems Checklist (CPC). 

*The ESAS-r* is a validated 9-item PRO measure of prevalent evidence-based symptoms experienced by patients with cancer and is a widely used instrument. Patients rate each symptom on a severity scale from 0 to 10, with 10 indicating the highest severity. The specific symptoms include pain, tiredness, drowsiness, nausea, lack of appetite, shortness of breath, anxiety, depression, and well-being [18,19]. 

*The symptom complexity score* in this study was derived from ESAS-r only. After review of our data, there was approximately 50% of the CPC questionnaire that was left blank. Therefore, we used a modified algorithm to account for this missing data. In a prior study, this modified algorithm was evaluated for sensitivity, specificity, and overall accuracy with the omittance of the CPC data [17]. There was negligible difference when the CPC data was omitted, and therefore, the ESAS-r was used only given its higher rate of completion. The ESAS-r considers the unique combination of symptoms and concerns the patient has self-identified. It rates the self-reported severity of symptoms and number of concerns indicated at a single visit and assigns a symptom complexity score (low, moderate, or severe) for the encounter. The ESAS-r symptom scores were the focus of this study. Patients were defined as having a *high symptom complexity* score if any one of the criteria were met: (1) any symptom scored 10 out of 10; (2) pain scored between 7 and 9; (3) 3–5 symptoms scored between 7 and 9; or (4) 6 or more symptoms scored between 4 and 6. A fifth group was derived for the purpose of this study to identify patients with multiple criteria; for example, tiredness was scored 10 (satisfies group 1), and pain was scored 8 (satisfies group 3) at the same visit [20]. 

### 2.4. Data Collection

A data abstraction form was created by the research study team, which included radiation oncologists and health service researchers. The abstraction form was trialled several times by two independent investigators (DY and PG). Discrepancies were discussed by the entire team until consistency and accuracy of data items to be collected were achieved. 

The abstractor accessed each patient’s electronic medical record to complete the abstraction form. We collected data from various sources within the electronic medical records, including any radiation healthcare professional’s (HCP) consultation or treatment progress notes, as well as treatment summary records and electronic orders.

The data was collected from two different sources, the ACR and EMRs. Unique patient identifiers, appointment type, and tumour type were collected from the provincial ACR. The remainder of the variables in the abstraction form, including demographic variables (age, sex) and cancer characteristics (cancer type, stage, treatment intent, type of treatment, treatment completion, and radiation appointment types), were collected from the chart review. Cancer types were categorised as breast, central nervous system (CNS), gastrointestinal (GI), genitourinary (GU), gynaecology (Gyne), head and neck (HN), haematology (Hem), and lung.

The ESAS-r symptom that had the highest score or the patient identified as the highest priority was selected as the main symptom per patient per appointment. This symptom was determined hierarchically either by (1) the HCP documenting the symptom as the main symptom during that appointment, (2) the symptom had the highest score reported by the patient, or (3) the symptom received the most attention during the appointment as determined by documentation in the patient’s electronic medical chart. For the main symptom of interest, the score was documented as well as the specific criteria met for the patient having been deemed high symptom complexity (criteria 1 through 5 as described above). 

Regarding the main symptom of interest, we abstracted data on whether the healthcare professional acknowledged the high symptom score (yes/no), assessed it (yes/no), and offered any intervention (yes/no). If an intervention was offered, we recorded the type of intervention and whether the patient accepted the intervention. The interventions were classified into the following categories: (1) medications, (2) referrals, (3) further investigations, (4) lifestyle modifications, (5) hospital admission, (6) NG tube, and (7) hydration. Referrals were further categorised into psychosocial, allied health, palliative care, and others. In addition to abstracting the notes from the visit consultation/progress note, we reviewed the additional comments and notes section within the PRO document for further referral details. The HCPs’ actions during the appointment were captured, including whether information/education was provided to the patient, if emotional support was offered, and whether a referral was offered and if it was accepted/declined by the patient. The best source of information was also collected (physician, nurse, or allied health note), as determined by the abstractor. Furthermore, for the additional symptoms that were endorsed, the same process of any assessment or interventions offered was documented. If there was specific documentation that the patient declined/did not complete the intervention, it was coded as “no”; otherwise, it was implied the patient had accepted the intervention.

Regarding all symptoms reported, the total number of high-intensity symptoms (score between 7 and 10) reported on each ESAS-r was documented, as well as the average number of high-intensity symptoms.

### 2.5. Analysis

#### 2.5.1. Descriptive Statistics

Demographic data and symptom outcomes were summarised using descriptive statistics. We compared the symptoms triggering a high symptom complexity score by tumour group and appointment type. We also compared the symptom management strategies and interventions by tumour group and appointment type. 

We described the number of patients who met multiple criteria (group 5) for a high symptom complexity score, along with the average number of high-intensity symptoms per tumour group. The top 3 symptoms were identified and compared across tumour groups and intervention types. We further described referral type by symptom, and the declined referrals by tumour group. 

#### 2.5.2. Logistic Regression

A multivariable regression model was completed for the primary outcome of whether an intervention was offered by a healthcare professional for the main high-intensity symptom. Covariates included in the model were age, sex, tumour type, appointment type (consultation, treatment review), symptoms (appetite, depression, nausea, pain, shortness of breath, tiredness, other), symptom severity score (≤6, 7, 8, 9 and 10), and treatment intent (palliative, curative). A significance level of 5% was used for all statistical tests. Data was exported into RStudio software, 2022.12.0+353, for analysis, and statistical significance was set a priori at *p* < 0.050. 

## 3. Results

### 3.1. Demographics

A total of 200 patients were included in the analysis. The average age across all tumour groups was 61.7 years, with 106 (53.0%) being female sex. One-half (50.5%) of patients received curative intent radiation, and the remainder received palliative intent radiation (49.5%). Forty-three percent of patients (*n* = 86) were seen for a consult appointment, and the remainder were seen for treatment review appointments while on active radiotherapy. Demographic data with the breakdown by tumour site are shown in Table 1. 

### 3.2. Descriptive Analysis

All tumour groups had patients who met multiple criteria for a high symptom complexity score and were assigned to group 5 (*n* = 149). The average number of high-intensity symptoms across all tumour groups was 3.3. The proportion of patients with high symptom complexity scores meeting multiple criteria (group 5) and the average number of high-intensity symptoms by tumour group and overall are shown in Table 2. 

### 3.3. Symptom Burden

**Figure 1:**
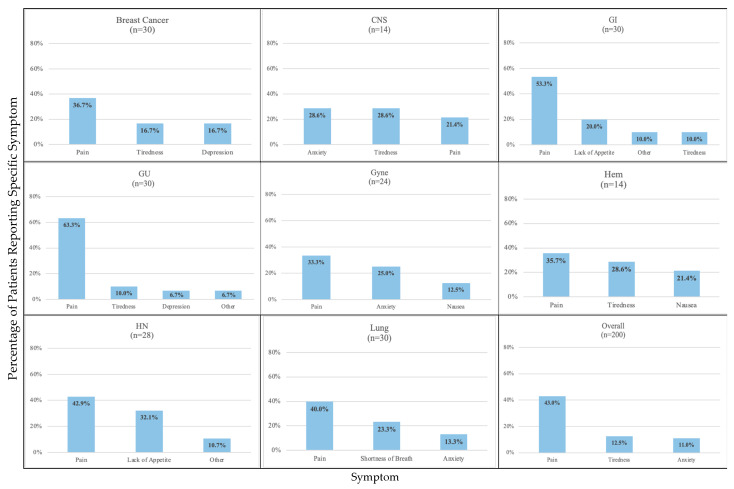
The top 3 high complexity symptoms by tumour group and overall.

For the specific symptoms experienced by patients, pain (43.0%) was reported most frequently as the main symptom, followed by tiredness (12.5%), anxiety (11.0%), other (9.5%), lack of appetite (9.0%), shortness of breath (5.0%), nausea (4.5%), depression (4.0%) and drowsiness (1.5%). The category of ‘other’ symptoms included bowel and bladder issues, cough, dysphagia, hot flashes, mood swings, new lump, skin, sleep, and taste issues. The breakdown across tumour groups found that the highest proportion of patients reporting pain was in patients with GU (63.5%) and GI (53.3%) cancers and the lowest in patients with CNS (21.5%) cancer. Tiredness was highest in patients with CNS (28.6%) and Hem (28.6%) cancers and lowest in patients with HN cancer (8.3%). Patients with CNS (28.6%) and Gyne cancers (25.0%) had anxiety reported most frequently as the main symptom, whereas patients with GI cancer did not identify anxiety as the main symptom. The top three high-intensity symptoms reported per tumour group are seen in Figure 1 and Table 2. 

### 3.4. Symptom Acknowledgement and Assessment

When reviewing the PPF, HCPs identified the main symptom 56.0% of the time and acknowledged the symptom with the patient 87.0% of the time. Across different tumour groups, the main symptom was acknowledged every time by HCPs in patients with HN cancer. Patients with CNS cancers had the lowest level of acknowledgement by HCPs (71.4%). 

### 3.5. Symptom Intervention

In total, 150 (75.0%) patients were offered an intervention for their main symptom. These interventions included medications (58.0%), referrals (40.7%), additional investigations (11.3%), lifestyle modifications (5.3%), hospital admission (3.3%), NG tube (2.0%), and hydration therapy (1.3%). Of those who were offered an intervention, 93.3% (*n* = 140) accepted the intervention. Pain was intervened on most frequently, followed by anxiety, lack of appetite, ‘other’, tiredness, nausea, shortness of breath, and depression. Drowsiness did not have any interventions offered. 

### 3.6. Interventions Offered by Symptom Type

**Figure 2:**
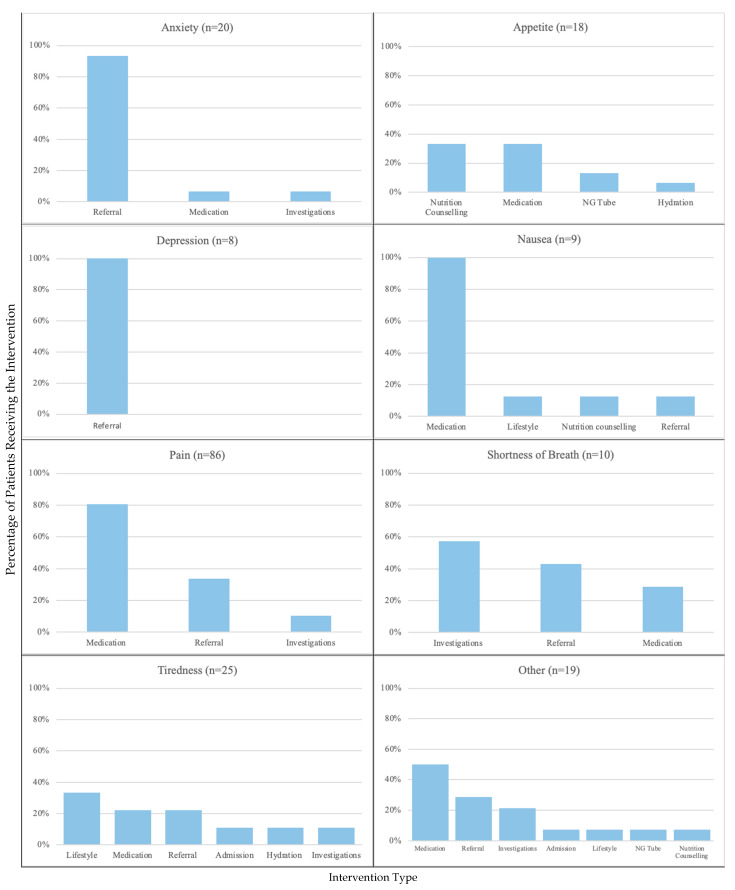
Types of interventions offered by symptom.

**Figure 3:**
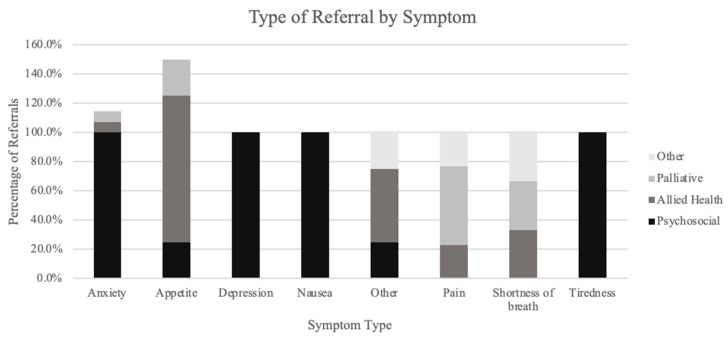
Type of referrals made by symptom type. For percentage of referrals >100%, patients may have had more than one type of referral offered for a symptom.

Pain was the most frequently reported symptom, and the most common intervention offered for pain was medication (80.5%), followed by referrals (33.8%) and investigations (10.4%). Of those referrals for pain, 53.8% were to palliative care. The most frequent intervention offered for tiredness was lifestyle modifications (33.3%). For anxiety, referrals were the most common intervention offered (93.3%). Appetite had nutrition counselling and medication referrals offered in the same proportions (33.3%). The only symptom with no interventions offered was drowsiness. Figure 2 shows the interventions offered by symptom type for each symptom. The most common referral type was psychosocial (41.0%); however, the type of referral varied depending on the symptom being attended to, as demonstrated in Figure 3. 

### 3.7. Interventions Declined

**Figure 4:**
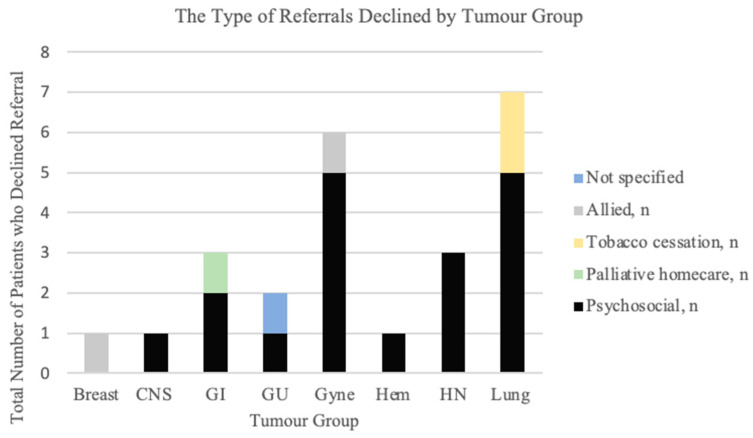
The type of referrals declined by tumour group.

Of the 150 patients offered an intervention, 6.7% (*n* = 10) patients declined the intervention. Patients with Gyne cancer declined interventions most often, followed by patients with lung and GU cancers. The types of interventions declined were referrals (*n* = 9), investigations (*n* = 2), and medications (*n* = 2). Referrals were declined most often by patients with lung cancer (23.3%), with the majority of the declined referrals being psychosocial (71.4%) and tobacco cessation support (28.6%). Patients with Gyne cancer declined referrals 20.8% of the time, with all referrals being for psychosocial support (Figure 4).

### 3.8. Logistic Regression

The multivariable regression model demonstrated factors associated with being offered an intervention (Table 3). Significant factors included a symptom score of 9 (OR = 9.56, 95% CI 1.64–62.84) and 10 (OR = 7.90, 95% CI 1.69–38.18) compared to symptom score of ≤6; palliative intent radiation treatment compared to curative intent (OR = 3.87, 96% CI 1.46–11.06); and last review appointment compared to consultation (OR = 1.93, 95% CI 0.68–5.82). When compared to tiredness, symptoms associated with being offered an intervention included pain (OR = 22.57, 95% CI 6.47–91.14), nausea (OR = 15.69, 95% CI 1.51–412.4), shortness of breath (OR = 7.97, 95% CI 1.20–63.74), and anxiety (OR = 6.69, 95% CI 1.58–31.64). Symptoms that were not associated with being offered an intervention were a lack of appetite and depression. Age, sex, and tumour site had no association with being offered an intervention. A summary of the logistic regression model can be seen in Table 3. 

## 4. Discussion

This study evaluated specific symptoms reported by patients with cancer seen in the radiation department who were noted to have a high symptom burden. The most common symptoms across all tumour groups were pain, tiredness, and anxiety (43.0%, 12.5%, and 11.0%, respectively). However, each tumour group showed variability in the top three symptoms experienced. 

Main symptoms were acknowledged and assessed by HCPs frequently (87.0%), and three-quarters of patients were offered an intervention for their main symptom. Most of the interventions offered were medications and referrals to interdisciplinary teams and specialists. The symptoms that were more likely to be offered an intervention included pain, anxiety, nausea, and shortness of breath. Palliative intent treatment and higher symptom scores of 9 and 10 were associated with being offered an intervention. 

Pain management is an integral part of symptom management in patients with cancer, with approximately half of patients experiencing pain throughout their disease course [21]. Our study found that almost half of patients reported pain as the main symptom. Patients undergoing palliative intent radiotherapy were more likely to be offered an intervention for their main symptom than those undergoing curative treatment. In 1991, the ESAS tool was originally developed for palliative care settings to provide regular assessments of symptoms to decrease the level of patient distress and suffering [18]. It has been recommended that patients diagnosed with metastatic disease or with high symptom burden have palliative services introduced in the early stages of their diagnosis [22,23]. Overall, symptom management in palliative patients is often well recognised as an area of focus. This is consistent with the results of the present study. Not surprisingly, patients undergoing palliative radiation treatment had interventions offered for their symptoms. A survey of practicing radiation oncologists on their comfort in palliative and supportive care management by Wei and colleagues showed that radiation oncologists were “moderately to very confident” in assessing pain (95.7%) and managing somatic pain (91.6%) and gastrointestinal symptoms (82.3%). However, they reported less confidence in the management of symptoms of anorexia (43.6%), anxiety (49.3%), and depression (33.7%) [24]. When comparing to our results, pain was the symptom with the most frequently offered intervention and depression less frequently, which may be in part related to the confidence of the HCPs in managing these symptoms. 

We found that anxiety was associated with being offered an intervention; however, depression was not. This was unexpected, as we had hypothesised that depression would have been associated with being offered an intervention and is closely related to anxiety. It is possible that patients were experiencing other physical symptoms during their appointment visit that were prioritised as the main symptom. A study of 124 radiation oncology adult patients found that 15% of patients reported significant depressive symptoms [25]. Furthermore, the somatic symptoms of depression overlap with the side effects of radiation treatment, including loss of energy, tiredness, and fatigue, and therefore, depression may not have been immediately identified as the main symptom during that visit [25].

Surprisingly, our model did not find an association with individual tumour type and whether an intervention was offered. However, our cohort of patients was selected to have high symptom complexity, and the previous literature reported that patients with higher ESAS scores for pain or shortness of breath were more likely to have specific symptom interventions taken in both breast and lung cancer tumour groups [26]. Furthermore, the symptom complexity algorithm and score were initially developed to flag attention to the clinical team if patients experience a high level of severe, concurrent symptom burden so that targeted care could be delivered [20]. Given this, it is likely that the high complexity symptoms were a greater factor in being offered an intervention than tumour group, given that the symptoms were more severe across all tumour groups. 

An interesting tumour-specific finding did show that across tumour groups, patients with lung cancer had the highest proportion of declining referrals, specifically for psychosocial or tobacco cessation. Similar studies have also found that patients with lung cancer are generally less likely to engage in supportive resources compared to other tumour groups [27,28,29].

### Strengths and Limitations

Our study focused intentionally on patients with high symptom burden determined through the evaluation of the self-reported symptoms in the ESAS-r. Given their higher degree of symptom burden, they require close assessment and intervention. Therefore, our results are most generalisable to outpatients receiving radiation treatment and experiencing high symptom burden, and less so to patients with cancer admitted as inpatients or undergoing systemic therapy alone. 

Additionally, our cohort included 200 patients in total, with the goal of having even representation across all tumour groups. However, some tumour groups, such as haematological and CNS malignancies, have fewer patients compared to the other tumour groups and may be less generalisable to these tumour groups. Overall, future research with a larger sample size and data is warranted to provide a more reliable and precise estimate of the relationships between predictor variables and the outcome.

The present findings that depression and lack of appetite are less likely to be offered an intervention identify a gap where care could be improved. This may include a specific focus on these symptoms during subsequent visits, a more integrated multidisciplinary approach at visits with allied healthcare providers, including dietician and psychosocial supports, and improved patient education materials, particularly for lack of appetite. Also, importantly, liaising with patients’ primary care providers to help manage these symptoms in the community. 

Despite these limitations, our study had many strengths. In our study, we included patients at different stages throughout their treatment journey, whether at the initial consultation or the completion of their treatment. This permitted broader generalisations over the entire treatment experience and not just isolation to a specific visit during their treatment.

Furthermore, our study cohort was very comprehensive. We included all different stages, both palliative and curative intent treatments, and we included the eight most common tumour groups. This provided a more comprehensive understanding of symptom type and intervention practices across the majority of tumour groups, compared to previous studies assessing individual or selective tumour groups. Overall, this is more reflective of a radiation oncology practice at a large tertiary radiotherapy centre. 

## 5. Conclusions

This is the first study to our knowledge that assesses the association of specific factors in patients with cancer and with high symptom burden undergoing radiotherapy with being offered an intervention for their symptoms. Our study unveiled that a significant proportion, over three-quarters of patients with high symptom burden, are offered an intervention for their main symptom and identified factors associated with being offered an intervention. By using this knowledge, we can better guide our clinical practice and patient care, with particular focus on symptoms of depression and lack of appetite, which are less likely to be offered an intervention. 

## Figures and Tables

**Table 1:** Descriptive data with the mean age and the frequencies of individual tumour groups and overall.

Sample Characteristics	Breast	CNS	GI	GU	Gyne	Hem	HN	Lung	Total
*N* total	30	14	30	30	24	14	28	30	200
Age (yrs)	60.7	47.4	64.0	71.0	59.7	63.8	60.0	66.6	61.7
Sex									
Female, n (%)	30 (100%)	11 (78.6%)	7 (23.3%)	3 (10.0%)	24 (100%)	9 (64.3%)	8 (28.6%)	14 (46.7%)	106 (53.0%)
Male, n (%)	0 (0.0%)	3 (21.4%)	23 (76.7%)	27 (90.0%)	0 (0.0%)	5 (35.7%)	20 (71.4%)	16 (53.3%)	94 (47.0%)
Cancer stage									
Stage 1	6 (20.0%)	4 (28.6%)	1 (3.3%)	1 (3.3%)	6 (25.0%)	1 (7.1%)	11 (39.3%)	4 (13.3%)	34 (17.0%)
Stage 2	6 (20.0%)	4 (28.6%)	4 (13.3%)	3 (10.0%)	7 (29.2%)	4 (28.6%)	3 (10.7%)	2 (6.6%)	33 (16.5%)
Stage 3	6 (20.0%)	3 (21.4%)	14 (46.7%)	7 (23.3%)	4 (16.7%)	2 (14.3%)	6 (21.4%)	3 (10.0%)	45 (22.5%)
Stage 4	12 (40.0%)	3 (21.4%)	11 (36.7%)	19 (63.3%)	7 (29.2%)	7 (50.0%)	8 (28.6%)	21 (70.0%)	88 (44.0%)
Treatment Intent									
Curative	18 (60.0%)	11 (78.6%)	10 (33.3%)	10 (33.3%)	16 (66.7%)	7 (50.0%)	22 (78.6%)	7 (23.3%)	101 (50.5%)
Palliative	12 (40.0%)	3 (21.4%)	20 (66.7%)	20 (66.7%)	8 (33.3%)	7 (50.0%)	6 (21.4%)	23 (76.7%)	99 (49.5%)
Appointment Type									
Consult	15 (50.0%)	5 (35.7%)	22 (73.3%)	21 (70.0%)	7 (29.2%)	1 (7.1%)	0 (0.0%)	15 (50.0%)	86 (43.0%)
First review	9 (30.0%)	6 (42.9%)	3 (10.0%)	5 (16.7%)	11 (45.8%)	6 (42.9%)	6 (21.4%)	9 (30.0%)	55 (27.5%)
Last review	6 (20.0%)	3 (21.4%)	5 (16.7%)	4 (13.3%)	6 (25.0%)	7 (50.0%)	22 (78.6%)	6 (20.0%)	59 (29.5%)
Treatment Receiving									
New Patient (pre-treatment)	15 (50.0%)	5 (35.7%)	22 (73.3%)	21 (70.0%)	7 (29.2%)	1 (7.1%)	0 (0.0%)	15 (50.0%)	86 (43.0%)
Radiation	12 (40.0%)	5 (35.7%)	2 (6.7%)	9 (30.0%)	10 (41.7%)	8 (57.1%)	16 (57.1%)	9 (30.0%)	71 (35.5%)
Chemoradiation	3 (10.0%)	4 (28.6%)	6 (20.0%)	0 (0.0%)	7 (29.2%)	5 (35.7%)	12 (42.9%)	6 (20.0%)	43 (21.5%)

Note: WHO staging was used for CNS patients. CNS = Central Nervous System, GI = Gastrointestinal, GU = Genitourinary, Gyne = Gynaecological, Hem = Haematological, HN = Head and Neck.

**Table 2:** The proportion of patients who met multiple criteria for high symptom complexity and the average number of high-intensity symptoms per tumour group and overall. The top 3 symptoms with proportions per tumour group.

Tumour Group	Number of Patients Who Met Multiple Criteria for HSC (Group 5)	Average Number of High-Intensity Symptoms	Top 3 Symptoms
Breast (*n* = 30)	22 (73.3%)	2.9	Pain (36.7%) Tiredness (16.7%) Depression (16.7%)
CNS (*n* = 14)	9 (64.3%)	2.9	Anxiety (28.6%) Tiredness (28.6%) Pain (21.4%)
GI (*n* = 30)	19 (63.3%)	3.3	Pain (53.3%) Lack of Appetite (20.0%) Other (10.0%) Tiredness (10.0%)
GU (*n* = 30)	21 (70.0%)	2.5	Pain (63.3%) Tiredness (10.0%) Depression (6.7%) Other (6.7%)
Gyne (*n* = 24)	20 (83.3%)	3.1	Pain (33.3%) Anxiety (25.0%) Nausea (12.5%)
Hem (*n* = 14)	10 (71.4%)	3.7	Pain (35.7%) Tiredness (28.6%) Nausea (21.4%)
HN (*n* = 28)	23 (82.1%)	3.7	Pain (42.9%) Lack of Appetite (32.1%) Other (10.7%)
Lung (*n* = 30)	25 (83.3%)	3.9	Pain (40.0%) Shortness of Breath (23.3%) Anxiety (13.3%)
Overall (*n* = 200)	149 (74.5%)	3.3	Pain (43.0%) Tiredness (12.5%) Anxiety (11.0%)

HSC = high symptom complexity. High Symptom Complexity score criteria: Group 1: Any symptom with a score of 10; Group 2: Pain scored 7–9; Group 3: 3-5 symptoms scored 7–9; Group 4: 6+ symptoms scored 4–6; Group 5: More than one criterion met. Example: A patient with breast cancer reports tiredness as a score of 10 (reaches criteria for HSC with group 1) AND scores pain an 8 (reaches criteria for HSC with group 3). Therefore, the patient has met more than 1 criterion and would be classified as Group 5.

**Table 3:** Logistic regression for patients who were offered an intervention for their main complex symptom. Covariates in model: age, gender, appointment type, tumour type, symptom score, and main symptom.

Covariates			Confidence Intervals		
	Univariate OR	Multivariate OR	LCL	UCL	*p*-Value
**Age, years**	0.999	0.986	0.949	1.02	0.447
**Female (ref.)**					
Male	1.46	0.923	0.298	2.74	0.886
**Consultation (ref.)**					
First review	1.48	1.93	0.675	5.83	0.228
Last review	2.24	6.22	1.84	23.3	0.00457 *
Intent palliative (ref. curative intent)	2.09	3.87	1.46	11.1	0.00843 *
**Breast (ref.)**					
CNS	1.20	2.30	0.398	14.1	0.354
GI	2.19	1.62	0.291	9.69	0.587
GU	2.67	2.75	0.496	16.1	0.251
Gyne	2.53	2.22	0.482	11.3	0.314
HN	8.67	2.98	0.392	30.7	0.313
Hem	0.889	0.358	0.057	2.18	0.264
Lung	2.19	1.20	0.244	6.25	0.821
**Symptom Score 6 (ref.)**					
7	2.09	3.46	0.727	16.9	0.118
8	2.44	4.18	0.912	19.6	0.0648
9	4.08	9.56	1.64	62.8	0.0144 *
10	3.85	7.80	1.69	38.2	0.00920 *
**Tiredness Main Symptom (ref.)**					
Anxiety	3.81	6.69	1.58	31.6	0.0123 *
Appetite	8.89	4.61	0.779	33.0	0.104
Depression	2.96	3.73	0.518	31.5	0.201
Nausea	14.2	15.7	1.51	412	0.0410 *
Other	3.11	2.99	0.736	13.1	0.133
Pain	15.2	22.6	6.47	91.1	<0.00100 *
Shortness of breath	4.15	7.97	1.20	63.7	0.0374 *

LCL: lower confidence limit; UCL: upper confidence limit; * *p* < 0.050.

## Data Availability

The de-identified data presented in this study is available on request from the corresponding author.
